# Sex-specific prevalence and outcomes of frailty in critically ill patients

**DOI:** 10.1186/s40560-020-00494-9

**Published:** 2020-09-29

**Authors:** Erin Hessey, Carmel Montgomery, Danny J. Zuege, Darryl Rolfson, Henry T. Stelfox, Kirsten M. Fiest, Sean M. Bagshaw

**Affiliations:** 1 Department of Critical Care Medicine, Faculty of Medicine and Dentistry and Alberta Health Services–Edmonton Zone, University of Alberta, 8440-112 ST NW, Edmonton, T6G2B7 Canada; 2 Department of Critical Care Medicine, Cumming School of Medicine, University of Calgary and Alberta Health Services–Calgary Zone, Calgary, Canada; 3 Alberta Critical Care Strategic Clinical Network, Alberta Health Services, Alberta, Canada; 4 Division of Geriatric Medicine, Department of Medicine, Faculty of Medicine and Dentistry, University of Alberta, Edmonton, Canada; 5 Department of Community Health Sciences and O’Brien Institute for Public Health, Cumming School of Medicine, University of Calgary, Calgary, Canada

**Keywords:** Frailty, Sex, Female, Critical care, Mortality, Mechanical ventilation, Renal replacement therapy, Outcomes

## Abstract

**Background:**

The prevalence of frailty, an important risk factor for short- and long-term outcomes in hospitalized adults, differs by sex. Studies in critically ill adults have also found differences in mortality and organ support rates in males and females. The objective of this study was to determine if these observed differences in mortality and organ support rates can be explained by sex and frailty alone, or if the interaction between sex and frailty is an important risk factor.

**Methods:**

This is a retrospective multi-centre population-based cohort study of all adult patients (≥ 18 years) admitted to the seventeen intensive care units (ICUs) across Alberta, Canada, between 2016 and 2017. On admission, physicians assigned a Clinical Frailty Scale (CFS) score (1 = very fit, 9 = terminally ill) to all patients. Patients with missing CFS scores or who died within 24 h of ICU admission were excluded. Frailty was defined as CFS ≥ 5. Outcomes included all-cause hospital mortality, ICU mortality, and organ support rates. A propensity score for female sex was generated and 1:1 matching on sex was performed. Multivariable Cox regression or logistic regression, as appropriate, was performed to evaluate the association between sex, frailty, and the sex-frailty interaction term with outcomes.

**Results:**

Of 15,238 patients included in the cohort, after propensity score matching 11,816 patients remained (mean [standard deviation] age 57.3 [16.9]). In the matched cohort, females had a higher prevalence of frailty than males (32% vs. 27%, respectively) and higher odds of frailty (odds ratio [95% confidence interval (CI)] 1.29 [1.20–1.40]). Though females were less likely to receive invasive mechanical ventilation (hazard ratio [95% CI] 0.78 [0.71–0.86]), the interaction between sex and frailty (i.e., males and females with and without frailty) was not associated with differences in organ support rates. Receipt of dialysis and vasoactive support, as well as hospital mortality and ICU mortality were associated with frailty but were not associated with female sex or the interaction between sex and frailty.

**Conclusions:**

Although frailty and sex were individually associated with mortality and differences in organ support in the ICU, there does not appear to be a significant interaction between sex and frailty with regards to these outcomes.

## Background

Frailty is increasingly recognized as an important risk factor for worse short- and long-term outcomes and for greater health care service use in hospitalized patients [1–3]. Research has estimated that approximately one-third of critically ill patients are frail [1, 4, 5]. A higher prevalence of frailty in females has been found in both critically ill and non-critically ill populations [1, 2, 6–8]. A systematic review of community-dwelling adults found that although females had a higher prevalence of frailty, mortality rates were higher in males living with frailty compared to females [7]. Studies in critically ill patients have found that mortality and organ support rates differ in frail and non-frail patients as well as between females and males [1, 3, 9].

Though frailty is associated with increased mortality and health service use in critically ill adults, little is known about how these associations differ by sex. A better understanding of the relationship between sex, frailty, and outcomes in critically ill adults would help develop more targeted decision-making and risk stratification models.

The objective of this study was to determine if the observed differences in mortality and organ support rates can be explained by sex and frailty alone, or if the interaction between sex and frailty is an important risk factor. We hypothesized that mortality and organ support rates would differ by the interaction between sex and frailty. Specifically, that mortality and organ support rates would differ in females with frailty compared to males with frailty, and to males and females without frailty.

## Methods

### Design, setting, population

**Fig. 1:**
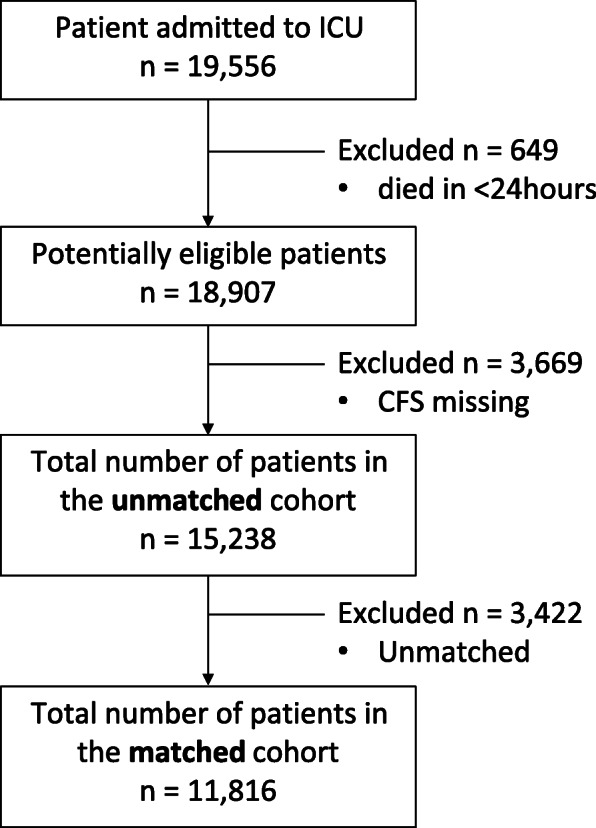
Patient selection. Abbreviation: ICU = intensive care unit; CFS = clinical frailty scale

This is a secondary analysis of a previously described retrospective multi-center population-based cohort [2]. All adult patients (age ≥ 18 years) admitted to any of the 17 intensive care units (ICUs; 14 mixed medical/surgical units; two cardiovascular surgical ICUs; one neurosciences ICU) in Alberta, Canada between 1 January 2016 and 30 June 2017 were eligible. These units are located in seven cities across Alberta: Edmonton (7 units); Calgary (5 units); Red Deer (1 unit); Lethbridge (1 unit); Grand Prairie (1 unit); Medicine Hat (1 unit), and Fort McMurray (1 unit), and comprise all units providing critical care services in the province. Patients missing a frailty score or who died within 24 h of ICU admission were excluded (Fig. 1). Approval from the Research Ethics Board at the University of Alberta, Edmonton was obtained (Pro00056591). Requirement for written informed consent was waived.

### Data source

The primary source of data was *eCritical Alberta*, a bedside clinical information system and data repository (*eCritical MetaVision*^*TM*^, iMDsoft, Germany; *eCritical TRACER*), described previously [2, 10]. Briefly, this database contains electronic ICU interdisciplinary clinical documentation including demographics, comorbidities, diagnostic classification, surgical status, illness severity scores (Acute Physiology and Chronic Health Evaluation [APACHE] II scores [11], Sequential Organ Failure Assessment [SOFA] scores [12]), and laboratory and intervention data (e.g., mechanical ventilation, vasoactive therapy, renal replacement therapy [RRT]). The *eCritical Alberta* system has been implemented in all ICUs in Alberta since 2012 and has a comprehensive quality assurance process to track and remediate completion of data elements. The data from *eCritical MetaVision*^*TM*^ is directly imported into *eCritical TRACER* using an extract-transform-load tool (Informatica, Redwood City, California). The *eCritical TRACER* repository is housed within Alberta Health Services (AHS) and has been extensively used for research, education, and planning and decision-making [10].

### Exposure and outcomes

Patient sex is coded in the *eCritical Alberta* system through linkage with the patient’s provincial health number (female = 0, male = 1; no missing data). No information about gender is available.

Frailty was defined using the Clinical Frailty Scale (CFS) score [13]. This is a validated 9-point score with 1 being very fit and 8 being severely frail (9 is terminally ill). This score was integrated into *eCritical MetaVision*^*TM*^ under the *Physician Admission Form* in December 2015. It is completed by the attending physician for every patient admitted to all adult ICUs in Alberta. Recent audit has shown compliance with completion of > 80% [2]. Frailty was defined as a CFS score ≥ 5 [2, 13].

Our outcomes included all-cause hospital mortality, ICU mortality, and organ support. Organ support measures were reported as binary (yes/no) variables and included invasive and non-invasive mechanical ventilation, vasoactive therapy, and RRT.

### Analysis

All analyses were performed on two cohorts: (1) unmatched and (2) propensity score matched. Baseline patient characteristics were stratified by sex. Continuous variables were compared using Mann-Whitney *U* test and binary variables were assessed using Chi^2^ test. Absolute standardized differences between males and females for each patient characteristic were calculated in both the matched and unmatched cohort.

In the unmatched cohort, the association between sex and frailty was evaluated using multivariable logistic regression. The association between sex, frailty and outcomes was assessed using Chi^2^ tests and by multivariable Cox regression or logistic regression, as applicable. The multivariable model included sex, frailty, age, APACHE II score, days between hospital and ICU admission, ICU diagnostic category, and an interaction term for sex and frailty status (i.e., male-frail; female-frail; male-non-frail; female-non-frail).

We then performed a propensity score-matched analysis. Propensity scores were computed for female sex using logistic regression. For the propensity score, we selected possible confounders that were associated with both sex and with a combined binary outcome measure (mortality and/or organ support yes/no) in univariate analysis with a level of significance of *p* < 0.01. The balanced propensity score (absolute standardized difference < 0.1) included age, admission diagnostic category (cardiac, gastrointestinal, respiratory, neurological, other), APACHE II score, and respiratory insufficiency on admission (from APACHE II chronic health problems, defined as severe COPD–requiring home oxygen, hypercapnia, or pulmonary hypertension). Frailty was excluded from the propensity score. Matches were made using nearest-neighbor matching with calipers (0.25*standard deviation) and no replacement (1:1 match) [14].

In the propensity score-matched sample, the association between sex and frailty was evaluated using logistic regression. The association between sex, frailty, and outcomes was assessed using multivariable Cox regression or logistic regression, as applicable, with a robust variance estimator used to account for the clustering within matched sets [15]. Frailty and an interaction term between sex and frailty were included in the multivariable model. The association between sex and outcomes was also evaluated in analyses stratified by frailty status. Kaplan-Meier mortality curves were produced for all-cause hospital mortality and ICU mortality stratified by sex and frailty in the matched sample and were censored at 90 days from admission. Stratified log-rank tests were used to evaluate associations [14, 15]. All analyses were conducted using STATA® version 12 (College Station, TX, USA).

## Results

### Patient population

A total of 15,238 patients were included in the unmatched cohort (5984 [39%] females, mean [standard deviation (SD)] age 57.9 [16.6] years). After propensity score matching, the total population was 11,816 patients (*n* = 5908 [99%] of females matched). Table 1 shows the patient characteristics stratified by sex and the absolute standardized difference before and after matching. Before matching, females were younger, had a higher illness severity score, and a higher prevalence of respiratory insufficiency on admission (Table 1). After matching, the standardized difference in all variables used to match was < 0.1.

**Table 1:** Comparison of patient characteristics by sex

	Unmatched				Matched		
	Male (*N* = 9254)	Female (*N* = 5984)	*p* value	Standardized difference	Male (*N* = 5908)	Female (*N* = 5908)	Standardized difference
Patient characteristics							
Age (mean [SD]), years	58.2 [16.4]	57.3 [17.0]	0.001	0.055	57.3 [16.7]	57.3 [17.0]	0.001*
ICU type			< 0.001				
Academic	4971 (54%)	2551 (43%)		0.227	2941 (50%)	2522 (43%)	0.143
Tertiary	1449 (16%)	1047 (18%)		0.053	999 (17%)	1042 (18%)	0.020
Community	1540 (17%)	1246 (21%)		0.107	1068 (18%)	1226 (21%)	0.069
Regional	1294 (14%)	1140 (19%)		0.137	900 (15%)	1118 (19%)	0.098
Admission category, (*n*;%)			< 0.001				
Medical/non-operative	5277 (57%)	3907 (65%)		0.172	3676 (62%)	3850 (65%)	0.061
Elective surgical	2353 (25%)	1094 (18%)		0.176	1222 (21%)	1084 (18%)	0.057
Emergency surgical	1576 (17%)	948 (16%)		0.004	967 (16%)	943 (16%)	0.011
No admission category assigned	48 (0.5%)	35 (0.5%)		0.008	43 (0.7%)	31 (0.5%)	0.029
Admission classification, (*n*;%)			< 0.001				
Medical	4154 (45%)	3352 (56%)		0.228	2902 (49%)	3308 (56%)	0.138
Neurology	690 (7%)	498 (5%)		0.031	512 (9%)	491 (8%)	0.013
Surgical	3796 (41%)	1954 (33%)		0.178	2091 (35%)	1935 (33%)	0.039
Trauma	556 (6%)	144 (2%)		0.180	354 (6%)	142 (2%)	0.180
No admission classification assigned	58 (0.6%)	36 (0.6%)		0.004	49 (0.8%)	32 (0.5%)	0.029
Diagnostic category, (*n*;%)			< 0.001				
Cardiovascular	3216 (35%)	1552 (26%)		0.194	1508 (26%)	1532 (26%)	0.009*
Respiratory	1956 (21%)	1531 (26%)		0.106	1495 (25%)	1514 (26%)	0.008*
Gastrointestinal/hepatic	1040 (11%)	717 (12%)		0.022	691 (12%)	706 (12%)	0.008*
Neurologic	1416 (15%)	1120 (19%)		0.091	1104 (19%)	1105 (19%)	0*
Other diagnosis	1504 (16%)	947 (16%)		0.010	993 (17%)	939 (16%)	0.025*
No diagnostic category assigned	122 (1%)	117 (2%)		0.051	117 (2%)	112 (2%)	0.007*
APACHE II score, (mean [SD])	18.4 [8.1]	19.0 [8.1]	< 0.001	0.074	19.0 [8.3]	19.0 [8.1]	0*
Admission SOFA score, (mean [SD])	6.5 [3.7]	6.1 [3.8]	< 0.001	0.114	6.5 [3.8]	6.1 [3.8]	0.116
Pre-ICU hospitalization duration (mean [SD]), days	4.1 [15.9]	3.7 [15.1]	< 0.001	0.023	3.7 [13.7]	3.6 [12.7]	0.010*
Frailty	2282 (25%)	1917 (32%)	< 0.001	0.164	1575 (27%)	1890 (32%)	0.117
CFS score (median [IQR])	3 [2–4]	4 [2–5]	< 0.001	0.147	3 [2–5]	4 [2–5]	0.122
APACHE chronic health condition variables							
Diabetes mellitus	2168 (23%)	1331 (22%)	0.09	0.029	1339 (23%)	1314 (22%)	0.010
Acute renal failure on admission	1802 (19%)	1210 (20%)	0.3	0.020	1244 (21%)	1199 (20%)	0.019
Respiratory insufficiency	950 (10%)	859 (14%)	< 0.001	0.125	827 (14%)	849 (14%)	0.011*
Congestive heart failure	746 (8%)	430 (7%)	0.05	0.036	468 (8%)	421 (7%)	0.030
Immunosuppression	660 (7%)	504 (8%)	0.003	0.049	459 (8%)	500 (8%)	0.026
Cirrhosis	389 (4%)	261 (4%)	0.6	0.008	264 (4%)	258 (4%)	0.005
Metastatic carcinoma	282 (3%)	186 (3%)	0.8	0	181 (3%)	181 (3%)	0
Hepatic failure	205 (2%)	166 (3%)	0.03	0.035	146 (2%)	163 (3%)	0.018
Lymphoma	81 (0.9%)	38 (0.6%)	0.1	0.027	51 (0.9%)	38 (0.6%)	0.025
Leukemia	77 (0.8%)	36 (0.6%)	0.1	0.025	45 (0.8%)	36 (0.6%)	0.018
Acquired immunodeficiency syndrome	37 (0.4%)	16 (0.3%)	0.2	0.023	25 (0.4%)	16 (0.3%)	0.026

*The variables in the propensity score. All standardized differences are absolute values

*Abbreviations*: *SD* standard deviation, *ICU* intensive care unit, *APACHE II* Acute Physiology And Chronic Health Evaluation II; *SOFA* The sequential organ failure assessment score, *CFS* clinical frailty scale, *IQR* interquartile range

### Association between sex and frailty

There was a higher proportion of females living with frailty prior to ICU admission (32%) compared to males (25%) (Table 1). Females also had a higher CFS score (median [interquartile range] females 4 [2–5] *vs.* males 3 [2–4], *p* < 0.001). Female sex was associated with higher odds of pre-admission frailty (CFS ≥ 5) in both the unmatched (adjusted odds ratio (OR) [95% confidence interval (CI)] 1.44 [1.34–1.56]) and matched cohorts (OR [95% CI] 1.29 [1.20–1.40]), respectively.

### Association between sex, frailty, and mortality

In the unmatched cohort, there was no difference in the prevalence of ICU or hospital mortality between males and females (Table 2). In multivariable analysis, neither sex nor frailty was associated with ICU mortality (Table 3). Frailty was associated with increased risk of hospital mortality (Table 3). The interaction between sex and frailty was not significant.

**Table 2:** Univariable analysis of the prevalence of outcomes stratified by sex in matched and unmatched cohort

	Unmatched		Matched	
	Male (***N*** = 9254)	Female (***N*** = 5984)	Male (***N*** = 5908)	Female (***N*** = 5908)
Any ventilation (invasive and non-invasive)	6858 (74%)	4128 (69%)**	4337 (73%)	4098 (69%)**
Invasive mechanical ventilation	6422 (69%)	3702 (62%)**	3996 (67%)	3676 (62%)*
Non-invasive ventilation	1039 (11%)	855 (14%)**	763 (13%)	850 (14%)*
Vasoactive support	4907 (53%)	2836 (47%)**	2922 (49%)	2817 (48%)
Renal replacement therapy	490 (5%)	313 (5%)	227 (6%)	313 (5%)
ICU mortality	767 (8%)	528 (9%)	527 (9%)	528 (9%)
Hospital mortality	1213/9177 (13%)	817/5939 (14%)	852/5857 (15%)	811/5866 (14%)

*p < 0.05

**p < 0.001

*Abbreviations*: *ICU* intensive care unit

**Table 3:** Multivariable analysis in matched and unmatched cohort

Variable	ICU mortality HR [95% CI]	Hospital mortality HR [95% CI]	Vasoactive support OR [95% CI]	Invasive mechanical ventilation OR [95% CI]	Renal replacement therapy OR [95% CI]	Non-invasive mechanical ventilation OR [95% CI]	Any ventilation (invasive or non-invasive) OR [95% CI]
Female sex							
Unmatched	1.03 [0.88–1.20]	1.01 [0.89–1.16]	0.88 [0.81–0.97]*	0.76 [0.70–0.84]*	0.92 [0.74–1.13]	1.08 [0.94–1.25]	0.77 [0.70–0.84]*
Matched	1.02 [0.87–1.19]	0.98 [0.85–1.12]	0.93 [0.86–1.02]	0.78 [0.71–0.86]*	0.91 [0.74–1.11]	0.99 [0.85–1.14]	0.76 [0.70–0.84]*
Frailty (CFS ≥ 5)							
Unmatched	1.04 [0.89–1.22]	1.31 [1.15–1.48]*	0.73 [0.65–0.82]*	0.54 [0.48–0.61]*	0.98 [0.78–1.23]	1.92 [1.65–2.22]*	0.71 [0.63–0.81]*
Matched	1.25 [1.05–1.49]*	1.61 [1.41–1.84]*	1.31 [1.17–1.47]*	0.78 [0.69–0.88]*	1.52 [1.21–1.92]*	2.78 [2.37–3.25]*	1.10 [0.96–1.25]
Sex-frailty interaction							
Unmatched	0.98 [0.77–1.25]	0.97 [0.80–1.17]	1.01 [0.85–1.20]	1.06 [0.90–1.26]	0.95 [0.67–1.33]	1.20 [0.97–1.48]	1.29 [1.07–1.55]*
Matched	1.00 [0.78–1.28]	0.97 [0.80–1.18]	0.95 [0.81–1.11]	1.06 [0.90–1.25]	0.99 [0.71–1.37]	1.17 [0.94–1.45]	1.24 [1.04–1.48]*

*p < 0.05

In the unmatched cohort, all results are adjusted for frailty, age, APACHE II score, days between hospital and ICU admission, ICU diagnostic category, and an interaction term for sex and frailty status. In matched analysis the models included sex, frailty, and the interaction term between sex and frailty

**Fig. 2:**
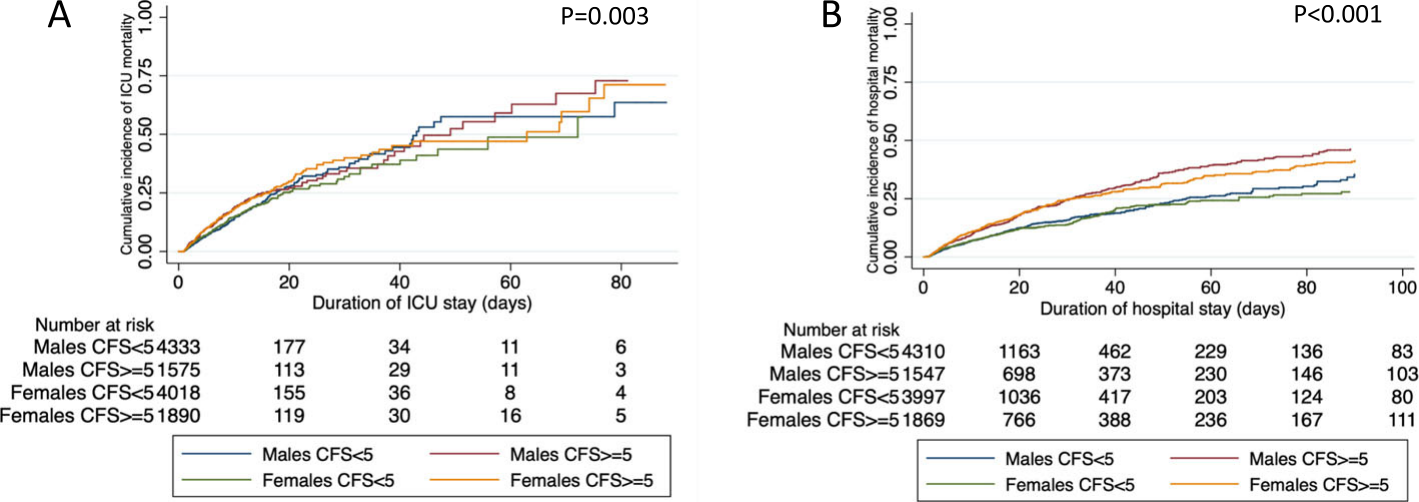
Kaplan-Meier curves for ICU and hospital mortality stratified by sex and frailty status. Kaplan-Meier curves evaluating the cumulative incidence of mortality stratified by sex and frailty status (CFS < 5 = non-frail; CFS > = 5 = frail) in the matched sample for **a** ICU mortality and **b** hospital mortality. *p* values calculated using stratified log-rank test, stratified on propensity score quintiles. There is a lower number of patients at risk in the hospital mortality curve because patients that were still in hospital at the time of data extraction were excluded from this analysis

In the matched cohort, ICU and hospital mortality did not differ between males and females (Table 2). After matching on important confounders, both ICU mortality and hospital mortality were associated with frailty (Table 3). There was no significant interaction between sex and frailty on mortality outcomes (Table 3). Similarly, in analyses only including patients with frailty, female sex was not associated with ICU or hospital mortality (adjusted hazard ratio [95% CI] 1.01 [0.84–1.22] and 0.95 [0.83–1.09], respectively). Kaplan-Meier survival curves stratified by sex and frailty show that both males and females with frailty had a higher cumulative incidence of ICU mortality, especially over the first 2 weeks of ICU admission, compared to patients without frailty (Fig. 2). Throughout the hospitalization, males and females with frailty also had higher cumulative incidence of hospital mortality compared with non-frail patients (Fig. 2).

### Association between sex, frailty, and organ supports

In the unmatched cohort, females received less vasoactive support and invasive mechanical ventilation, but more non-invasive ventilation compared to male patients (Table 2). In the multivariable analysis, both female sex and pre-admission frailty were associated with lower odds of receiving vasoactive support and mechanical ventilation (Table 3). Frailty was also associated with greater odds of receiving non-invasive ventilation. Receipt of RRT was not associated with sex or frailty (Table 3).

In the matched cohort, there was no longer a difference in the prevalence of vasoactive support between males and females (Table 2). However, female patients still had lower utilization of invasive mechanical ventilation, but higher utilization of non-invasive ventilation than male patients. In multivariable analysis, female sex and frailty were associated with lower odds of invasive ventilation (Table 3). Both frailty and female sex were individually associated with receiving less ventilation (non-invasive or invasive ventilation) overall; however, the sex-frailty interaction was associated with increased odds of receiving any ventilation (Table 3). Frailty was associated with higher odds of receiving vasoactive support, RRT, and non-invasive ventilation. The interaction between sex and frailty was not associated with non-invasive ventilation, invasive mechanical ventilation, vasoactive support, or RRT in the matched or unmatched cohorts (Table 3). In stratified analysis only comparing patients with frailty, females had lower odds of receiving mechanical ventilation (OR [95% CI] 0.83 [0.72–0.95]).

## Discussion

In this large multi-center population-based cohort study of adult patients admitted to ICUs across Alberta, Canada, we found that after propensity score matching female and male patients on important clinical variables, females had a higher prevalence and odds of having clinical frailty on ICU admission compared to males. Though frailty was associated with higher ICU and hospital mortality and differences in organ support rates, there was no interaction between sex and frailty with these outcomes. In the matched cohort, females received less invasive mechanical ventilation than male patients, but frailty did not appear to be an important effect modifier for this association.

We found that the interaction between frailty and sex was not associated with meaningful differences in ICU or hospital mortality. Frailty has been shown to be associated with increased risk of ICU and hospital mortality in meta-analysis [3]. In matched analysis, we similarly found that patients living with frailty were at higher risk of both ICU and hospital mortality. Previous research has also found sex differences in ICU mortality [9, 16, 17]. With females having a higher prevalence of frailty in the ICU, we aimed to assess if this interaction could explain the higher mortality rates seen in female patients [9, 17]. Based on our analysis, both females and males living with frailty had higher mortality than non-frail patients; however, in multivariable analysis, only frailty was independently associated with ICU mortality. This suggests that previous analyses showing differences in mortality by sex may have been confounded by frailty status which was not assessed but has been shown to be an important risk factor for mortality in this population [9, 16, 17]. Understanding that the association between frailty and mortality does not differ by sex is important for clinicians as it will allow for more specific risk stratification models and help guide patient-centered discussions and interventions.

A higher prevalence of frailty in females has been found in both ICU and community-based studies [1, 2, 6–8]. In the matched population with similar demographic and clinical variables, we found the prevalence of pre-admission frailty (CFS ≥ 5) in females was higher than in males (32% vs 27%), and that females had nearly 30% higher odds of having clinical frailty on ICU admission. Previous literature has hypothesized that sex differences in the prevalence of frailty may be partly attributed to females living longer and having greater multimorbidity [7, 8]. While these factors likely contribute, based on our matched cohort which had similar age and burden of comorbidities, these are not the only explanation for the higher prevalence of frailty in females. Other factors that contribute to the development of frailty include social and environmental factors such as marital status, household income, and education level [8, 18]. In the ICU population specifically, a Canadian study found that while females were more likely to be hospitalized, they were less likely to be admitted to the ICU [9]. It should be noted that this study did not look at differences in goals-of-care status and was not able to assess sex differences in the population that received an ICU consult but was not admitted to ICU. Furthermore, frailty was not assessed in this study, and it is unclear if this sex difference in admission to the ICU could play a role in the difference in prevalence of frailty by sex observed in critically ill patients. Another factor that could play a role is bias in the assessment of frailty using the CFS score (and threshold for frailty) on ICU admission. In this cohort, a CFS score was assigned on admission by the attending ICU physician. One small study found good inter-rater reliability using the CFS in ICU but it did not stratify the analysis by sex [19]. Unfortunately, in our database, we did not have access to social or gender factors that could help us further understand the sex difference in pre-admission frailty in ICU. However, we did show that sex-based frailty differences persisted even after matching for age, illness severity (APACHE II score), and in the absence of any differences in various comorbidities.

In males and females matched on important demographic and clinical characteristics, we found that females were less likely to receive invasive mechanical ventilation. This finding is similar to another Canadian study which found 58% of males compared to 52% of females received mechanical ventilation during their course in ICU [9]. Frailty was also associated with lower rates of invasive mechanical ventilation, but higher rates of vasoactive support, dialysis, and non-invasive ventilation. A meta-analysis by Muscedere et al. found no difference in rates of mechanical ventilation or vasoactive support in frail and non-frail patients [3]. Despite female sex and frailty both being associated with lower rates of mechanical ventilation, there was no difference in mortality outcomes when evaluating the interaction between sex and frailty. However, we found that when only evaluating patients with frailty, females had lower odds of receiving mechanical ventilation. Interestingly, there was no difference in respiratory diagnosis or respiratory insufficiency on admission between males and females receiving mechanical ventilation. In our cohort, we did not have access to information about goals-of-care prior to admission, or discussion or changes made during ICU admission, surrogate decision-makers, or other social factors that may have influenced the decision to provide or receive mechanical ventilation. Further research is needed to better understand these sex and frailty differences in organ support rates in ICU.

Our study has a number of strengths. This was a large multi-center population-based study which allowed us to perform propensity score-matched analysis to evaluate sex and frailty differences in mortality and organ support rates in ICU, which have been evaluated separately in previous studies. Our propensity score-matched analysis retained a large number of patients and significantly reduced the bias and standardized differences between male and female patients on important demographic and clinical characteristics. Frailty was measured by the attending physician prospectively upon ICU admission using a validated frailty measure rather than retrospective ascertainment [13].

Our study also has limitations. Decision-making for the initiation and for the withdrawal of life-sustaining therapies in the ICU is multifactorial. We attempted to balance the clinical factors that may have contributed to this decision-making process; however, we did not have information about gender, socio-economic status, goals-of-care, or other social factors that may have confounded this relationship. Although we were able to significantly decrease the standardized difference in many important confounders using propensity score matching, some variability still existed between male and female patients. These variables were unable to be balanced in the model and had to be excluded from the propensity score. As such, it is possible that there are some residual confounders we were unable to control for in our analysis. While we were able to control for respiratory admission category and pre-admission respiratory insufficiency, we did not have information about the nuance etiology of respiratory failure in patients receiving mechanical ventilation. Future research is needed to explore the association between sex and mechanical ventilation use in ICU. We do not have data on inter-rater reliability or bias in the assignment of CFS score by ICU physicians. We recognize that our study is a secondary analysis, and as such, no sample size calculation was performed and our cohort may have had limited statistical power.

## Conclusions

In a large cohort of critically ill patients matched on important demographic and clinical factors, we found that the prevalence of clinical frailty was higher in female compared to male patients. Although both frailty and sex have individually been associated with mortality and differences in organ support rates in ICU, the interaction between these two features does not appear to be an important risk factor for these outcomes. Rather, frailty is independently associated with increased mortality, increased use of vasoactive support, dialysis, and non-invasive ventilation, and with decreased use of mechanical ventilation. Female sex is independently associated with lower odds of mechanical ventilation in ICU, but not mortality. Overall, understanding the relationship between frailty, sex, and their interaction with outcomes may allow for more specific risk stratification models to be developed and help guide patient-centered care in the ICU. Further research is needed to explore whether sex differences in assigned frailty status are due to other social or biological factors or if they are due to bias in ICU admission selection or assessment of frailty status. Understanding the sex differences in organ support rates in ICU also requires further exploration into social or gender factors that could explain this association.

## Data Availability

The datasets generated and/or analyzed during the current study are not publicly available due ethics board regulations but may be available from the corresponding author on reasonable request.

## Abbreviations

OR: Odds ratio
HR: Hazard ratio
CI: Confidence interval
SD: Standard deviation
ICU: Intensive care unit
APACHE II: Acute Physiology And Chronic Health Evaluation II
SOFA: Sequential organ failure assessment
CFS: Clinical frailty scale
RRT: Renal replacement therapy
